# Somatostatin prevents lipopolysaccharide-induced neurodegeneration in the rat substantia nigra by inhibiting the activation of microglia

**DOI:** 10.3892/mmr.2015.3494

**Published:** 2015-03-13

**Authors:** LIJUAN BAI, XIQUE ZHANG, XIAOHONG LI, NA LIU, FAN LOU, HONGLEI MA, XIAOGUANG LUO, YAN REN

**Affiliations:** 1 Department of Neurology, The First Affiliated Hospital of China Medical University, Shenyang, Liaoning 110001, P.R. China; 2 Department of Neurology, The People’s Hospital of Liaoning Province, Shenyang, Liaoning 110016, P.R. China

**Keywords:** somatostatin, neuron, substantia nigra, microglia, Parkinson’s disease

## Abstract

Somatostatin (SST) is a neuromodulator which is abundant throughout the central nervous system (CNS) and has a crucial role in neurodegenerative disorders. However, little is known about the effects and mechanisms of SST in dopaminergic (DA) neurons in the context of Parkinson’s disease (PD). In the present study, a model of PD was generated by injecting lipopolysaccharide (LPS) into the substantia nigra (SN) of rats in order to investigate the effects of SST on LPS-induced degeneration of DA *in vivo*. Intramural injection of LPS resulted in a significant loss of DA neurons, while reduction of neuronal death by SST pretreatment was confirmed using immunohistochemical staining for tyrosine hydroxylase and Nissl. In parallel, immunohistochemical detection of OX-42 and hydroethidine staining were employed to determine the activation of microglia and production of reactive oxygen species (ROS), respectively. It was found that SST inhibited the LPS-induced microglial activity and ROS production. ELISA revealed a decreased production of pro-inflammatory mediators, including tumor necrosis factor-α, interleukin-1β and prostaglandin E2 when SST was administered prior to LPS treatment. Western blot analysis showed that LPS-induced expression of inducible nitric oxide synthase, cyclooxygenase-2 and nuclear factor κB (NF-κB) p-p65 was attenuated by administration of SST prior to LPS application. The results indicated that LPS-induced loss of nigral DA neurons was inhibited by SST and the observed effects of SST on neuroprotection were associated with suppression of microglial activation and the NF-κB pathway, ensuing decreases of neuroinflammation and oxidative stress. The present study therefore suggested that SST is beneficial for treating neurodegenerative diseases, such as PD, through inhibiting the activation of microglia.

## Introduction

Parkinson’s disease (PD) is a common neurodegenerative disease, leading to cell death of dopaminergic (DA) neurons in the substantia nigra (SN) (1). An approximate of 5–10% of PD can be attributed to heritable genetic mutations; however, the cause of more common PD and the mechanisms underlying the development of the disease have remained elusive (2,3). Several lines of evidence suggest that activated microglia have a critical role in the pathogenesis of PD through producing inflammatory mediators, including tumor necrosis factor (TNF)-α, interleukin (IL)-1β and neurotoxin, including inducible nitric oxide synthase (iNOS) and cyclooxygenase-2 (Cox-2) (4). Along with microglial activation, it has been reported that the production of reactive oxygen species (ROS) can accelerate the death of DA neurons (5). Increases of pro-inflammatory cytokines, activation of the nuclear factor (NF)-κB signaling pathway and oxidative stress were observed in the destroyed DA neurons (6). Therefore, all these factors can be regarded as potential targets for treating PD.

Somatostatin (SST) as an inhibitor of growth hormone (GH) has been identified to be abundant throughout the central nervous system (7). As a neuromodulator, SST has a crucial role in memory and cognition (8). In addition, the decreased SST levels in the frontal and entorhinal cortex as well as the hippocampus have been correlated to the cognitive deficits in patients with PD (9). However, increases in SST have been observed in the cerebrospinal fluid of patients with early PD (10). Due to the controversial data reported, the function and mechanism of SST which participates in PD are still subject to extensive examination. Accumulating clinical and experimental evidence suggested that SST can provide potential therapy in neurodegenerative disorders involving cognitive dysfunctions (11). To date, little is known about the effects of SST in DA neurons in the context of PD and its mechanism of action. To investigate this matter, an animal model of PD was generated by injecting lipopolysaccharide (LPS) into the SN of rat brains. It was examined whether SST administered prior to LPS treatment was able to protect nigral DA neurons from LPS-induced neurotoxicity through inhibiting microglial activation and reducing subsequent neuroinflammation as well as oxidative stress. To clarify the functional mechanism of SST, the expression of NF-κB p65 and downstream factors were investigated in the present study.

## Materials and methods

### Reagents

The following reagents and kits were used in the present study: Rabbit polyclonal anti-tyrosine hydroxylase (TH; cat. no. 2792; Cell Signaling Technology, Inc., Boston, MA, USA), rabbit polyclonal anti-OX-42 (cat. no. orb11009; Biorbyt, Cambridge, UK), hydroethidine (Molecular Probes, Eugene, OR, USA), LPS (Sigma-Aldrich, St. Louis, MO, USA), heparin (Qianhong Bio-pharma Co., Ltd., Changzhou, China), SST (ProSpec, East Brunswick, NJ, USA), biotinylated goat anti-rabbit secondary antibody (cat. no. A0277) and horseradish peroxidase (HRP)-labeled streptavidin (Beyotime, Shanghai, China), bicinchoninic acid (BCA) kit (Beyotime), Rat TNF-α ELISA kit, Rat IL-1β ELISA kit and Prostaglandin E2 ELISA kit (PGE2; USCN, Wuhan, Hubei, China).

### Stereotaxic surgery and SST administration

All experiments were performed according to the approved animal protocols and guidelines established by Chung *et al* (12). 144 female Sprague Dawley rats were provided by the Experimental Animal Centre of China Medical University (Shenyang, China). The animal study protocol was approved by the Animal Experimental Committee of China Medical University, and the mice received humane care according to the Principles of Laboratory Animal Care. They were randomly assigned to six experimental groups and received unilateral administration of 3 *μ*l phosphate-buffered saline (PBS), LPS (5 *μ*g in 3 *μ*l PBS), 2 *μ*l saline + LPS, 2 *μ*l SST (20 *μ*g/kg) + LPS, 2 *μ*l SST (40 *μ*g/kg) + LPS, 2 *μ*l SST (40 *μ*g/kg) respectively, into the right SN as previously described by Chung *et al* (10). 24 rats of each treated group were separated into three different subgroups for various analyses, including OX-42 and hydroethidine tests of brains after 24 h of treatment, western blot and ELISA assays of SN after 24 h of treatment, as well as immunohistochemical detection of TH and Nissl in the SN after 7 days of treatment. SST was injected 1 h prior to LPS treatment.

### Tissue preparation and immunohistochemistry

Brain tissues were prepared for immunohistochemical staining as previously reported (12). Tissues were dehydrated by using a graded ethanol series of 70% ethanol for 2 h, 80% overnight, 90% for 2 h and 100% for 2 h. Brain tissues were then post-fixed in dimethylbenzene (China National Medicines Corporation Ltd., Beijing, China) for 30 min and embedded in dimethylbenzene-paraffin at 60°C for 2 h, after which samples were embedded in a metal frame. Coronal sections (5 *μ*m) cut by a sliding microtome CM69001 (Leica Microsystems, Mannheim, Germany) were spread in warm water and placed onto glass slides to dry in a 70°C-chamber for 40 min. Sections were dewaxed using ethanol and then boiled in antigen retrieval solution for 10 min. The cooled sections were incubated in 3% H_2_O_2_ (China National Medicines Corporation Ltd.) for 15 min at room temperature and then blocked with normal goat serum (Beijing Solarbio Science & Technology Co., Ltd., Beijing, China) for 15 min. Sections were incubated overnight at room temperature with rabbit anti-OX-42 (1:100; cat. no. orb11009) for microglia and rabbit anti-TH (1:100; cat. no. 2792) for DA neurons. Following removal of unbound primary antibodies by washing, biotinylated goat anti-rabbit secondary antibody (1:200; cat. no. A0277) was added and incubated for 30 min at 37°C. Sections were then washed with PBS and incubated with HRP-labeled streptavidin for 30 min at 37°C. Finally, 100 *μ*l diaminobenzidine (Beijing Solarbio Science & Technology Co., Ltd.) was added for coloration and hematoxylin stain (Beijing Solarbio Science & Technology Co., Ltd.) for counterstaining. Hydroethidine (Molecular Probes) was used for *in situ* detection of O_2_^−^ and O_2_^−^ -derived oxidants. For Nissl staining, a number of the SN tissue samples were stained in 0.5% cresyl violet (China National Medicines Corporation Ltd.). Following washing with water and dehydrating with ethanol as well as treating with dimethylbenzene (China National Medicines Corporation Ltd.), stained samples were analyzed under a stereo microscope (BX51; Olympus Corporation, Tokyo, Japan) or viewed with a confocal laser scanning microscope (FV1000S-SIM/IX81; Olympus Corporation).

### Stereological estimation

The total number of TH-positive neurons was counted in the various groups at seven days post-injection (LPS, PBS, SST or a combination) using the stereo microscope BX51 (Olympus Corporation). This unbiased stereological method of cell counting according to a previously described method is not affected by either the counted elements (neurons) or the size of the reference volume (SN) (13).

### Western blot analysis

For western blot analysis, protein was extracted from the SN of eight rats from each group following 24 h of treatment. Following determination of the protein concentration using a BCA kit, 40 *μ*g protein was boiled in PBS with 5X loading buffer (Beijing Solarbio Science & Technology Co., Ltd.) for 5 min. The bands of protein separated by SDS-PAGE (8% gel for iNOS, 10% gel for NF-κB p65 and NF-κB p-p65, and 12% gel for Cox-2) were transferred onto polyvinylidene difluoride membranes (Millipore, Billerica, MA, USA) using an electroblot apparatus (DYCZ-40D, Beijing, China). Filters were blocked in 5% non-fat milk and incubated separately overnight at 4°C with the following primary antibodies: Rabbit polyclonal anti-iNOS (1:1,000; cat. no. bs-2072R; Bioss, Beijing, China), goat polyclonal anti-Cox-2 (1:100; sc-1747), mouse monoclonal anti-NF-κB p-p65 (1:100; cat. no. sc-166748) and rabbit polyclonal anti-NF-κB p65 (1:100; cat. no. sc-372) obtained from Santa Cruz Biotechnology, Inc. (Dallas, TX, USA). Membranes were then washed with Tris-buffered saline containing Tween-20 (TTBS) (BioSharp, Hefei, China) and incubated with the following corresponding HRP-conjugated secondary antibodies: Goat anti-mouse immunglobulin (Ig)G-HRP (1:5,000; cat. no. A0216), goat anti-rabbit IgG-HRP (1:5,000; cat. no. A0208) and donkey anti-goat IgG-HRP (1:5,000; cat. no. A0181) (Beyotime, Shanghai, China) for 45 min at 37°C. Following washing with TTBS, protein bands were visualized using enhanced chemiluminescence reagent (cat. no. E002-5; Qihai Biotec, Shanghai, China). Protein levels were quantified by gray value analysis using Gel-Pro-Analyzer (Media Cybernetics, Inc., Rockville, MD, USA).

### Measurement of TNF-α, IL-1β and PGE2

The production of TNF-α, IL-1β and PGE2 from the SN of rats was determined by ELISA. Proteins were extracted through homogenizing tissues and quantitated using the BCA kit. ELISA was then performed according to the manufacturer’s instructions.

### Statistical analysis

Values are expressed as the mean ± standard deviation. All raw data were analyzed by one-way analysis of variance followed by the Bonferroni test for post hoc comparisons. Statistical analyses were performed using GraphPad Prism 5.0 software (GraphPad Software Inc., La Jolla, CA, USA). P<0.05 was considered to indicate a statistically significant difference between values.

## Results

### Neuroprotective effect of SST on the LPS-treated SN

**Figure 1:**
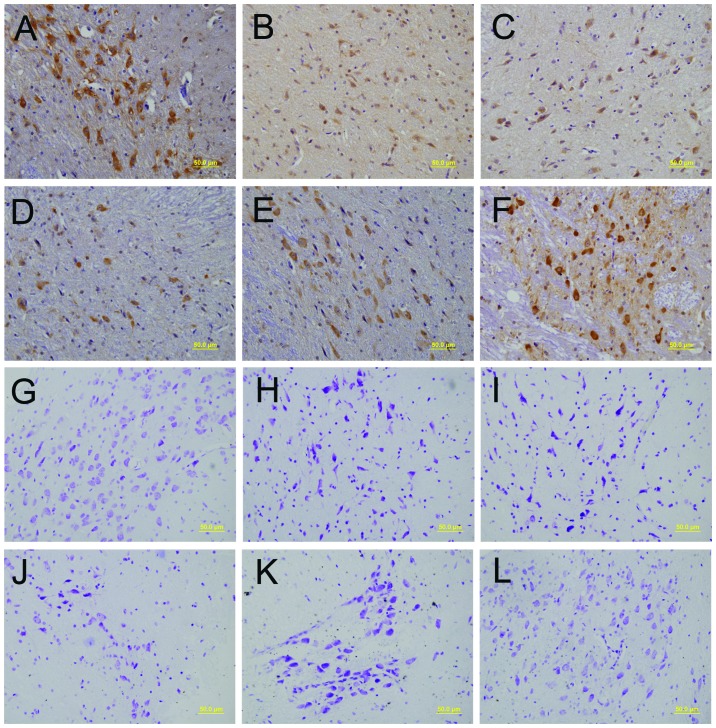
Effect of SST on LPS-induced loss of nigral dopaminergic neurons. Rats were sacrificed at the 7^th^ day after LPS injection, and their brains were selected for immunostaining with (A-F) tyrosine hyrdoxylase antibody and (G-L) Nissl staining. (A and G) Control treated with phosphate-buffered saline, (B and H) LPS model, (C and I) LPS + saline, (D and J) LPS + 20 g/kg SST, (E and K) LPS + 40 *μ*g/kg SST, and (F and L) 40 *μ*g/kg SST. Scale bars, 50 *μ*m. LPS, lipopolysaccharide; SST, somatostatin.

LPS injection into the SN was previously shown to result in a considerable loss of TH- and Nissl-positive cells, as well as alterations in the morphology of TH-positive neurons to shrunken neuronal cell bodies (12,13). To investigate the potential of SST to prevent LPS-induced neurotoxicity of nigral neurons, SST was administered 1 h prior to LPS injection. SST significantly attenuated the LPS-mediated loss of TH-positive DA neurons in the SN and preserved normal neuronal morphology, as evidenced by an increased number of healthy cell bodies and processes with enhanced staining intensity (Fig. 1A-F). As shown in Fig. 1G, the morphology of the neurons were integrative and well-maintained. In Fig. 1H, the number of healthy neurons was markedly decreased, and the number of dead neurons increased, as characterized by the chromatolysis of Nissl bodies and the presence of shrunken neuronal cell bodies with pyknotic nuclei surrounded by a thin band of cytoplasm. In Fig. 1K, the above pathological damages were notably reduced, following pretreatment with higher concentrations of SST. The results of the LPS + saline group (Fig. 1I) were similar to those exhibited in Fig. 1H. In addition, the results of the LPS + 20 g/kg SST group (Fig. 1J) were similar, but not as obvious, as those exhibited in Fig. 1K, and the results of the 40 *μ*g/kg SST group (Fig. 1L) were similar to the results shown in Fig. 1G, thus indicating that SST had no cytotoxic effects on the neurons. These results showed that SST had neuroprotective effects on LPS-treated SN.

### LPS-induced microglial activation is inhibited by SST

**Figure 2:**
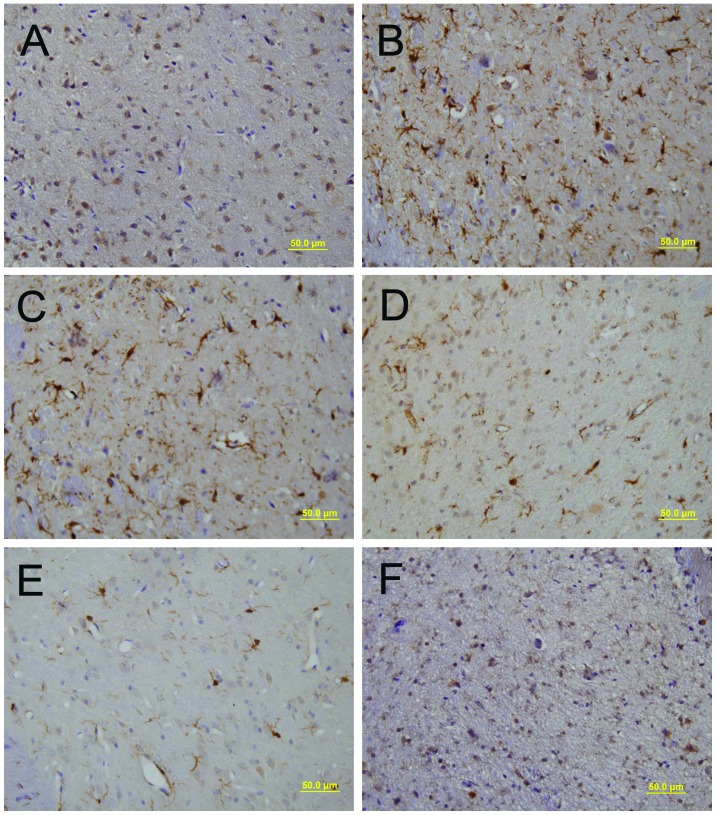
Effect of SST on LPS-induced microglial activation in the substantia nigra. Brains were immunostained with OX-42 antibody for ana-lyzing the expression of OX-42. (A) Control treated with phosphate-buffered saline, (B) LPS model, (C) LPS + saline, (D) LPS + 20 *μ*g/kg SST, (E) LPS + 40 *μ*g/kg SST, (F) 40 *μ*g/kg SST. Scale bars, 50 *μ*m. LPS, lipopolysaccharide; SST, somatostatin.

It has been reported that LPS is able to activate rat microglia and lead to neuronal death *in vivo* (14). Thus, the present study investigated the effect of SST on LPS-induced microglial activation in the SN. SN sections were prepared for immu-nohistochemical staining using antibodies against OX-42 to detect microglial activation. The majority of OX-42-positive microglia exhibited a resting morphology in the PBS-injected SN (Fig. 2A), whereas LPS-treated samples showed activated microglia with enhanced staining intensity and larger cell bodies with short, thick processes (Fig. 2B). Pre-treatment with SST dramatically decreased the number of activated microglia induced by LPS compared that in the saline-pre-treated control (Fig. 2C-E), while SST alone had no effect on microglial activation (Fig. 2F). These findings suggested that SST inhibited the LPS-induced activation of microglia.

### LPS-induced ROS production is inhibited by SST

**Figure 3:**
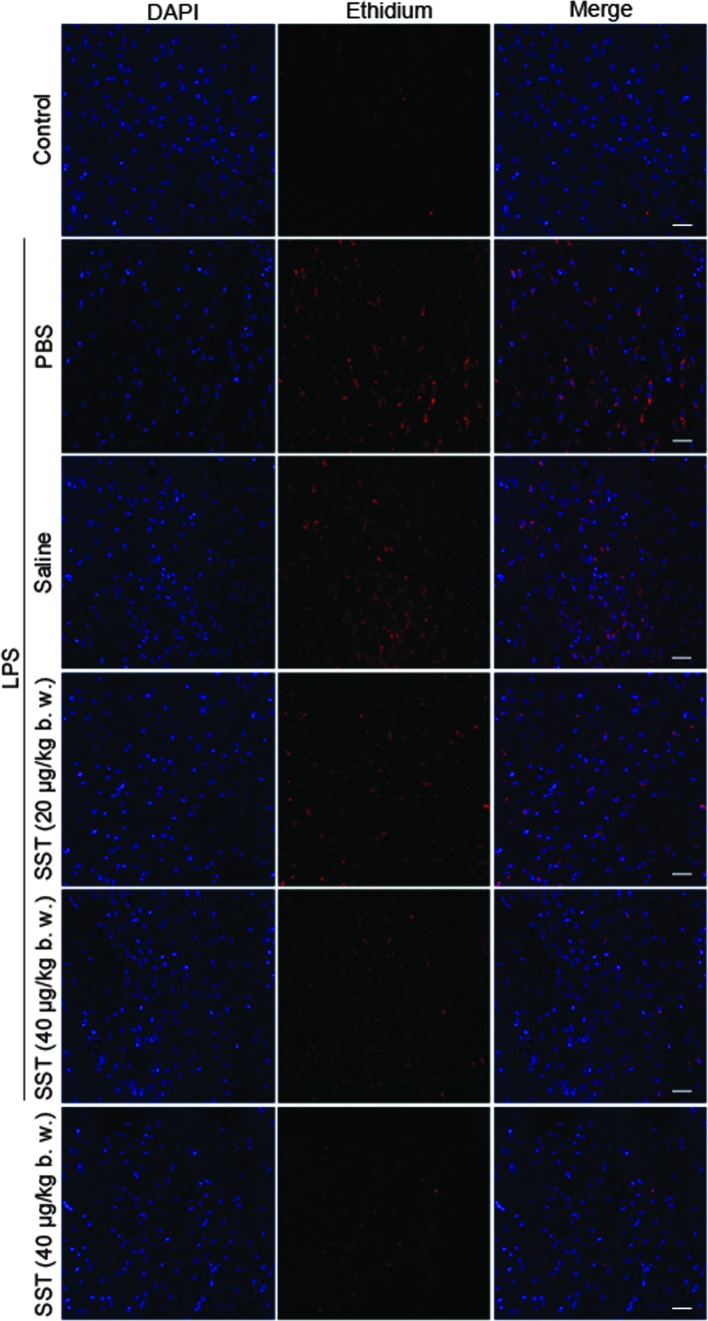
Effect of SST on LPS-induced production of reactive oxygen species. At 24 h following LPS injection in the presence or absence of SST, brains subjected to examination of the production of oxidized hydroethidine by testing the accumulated fluorescence through confocal laser scanning microscopy. Scale bars, 30 *μ*m. LPS, lipopolysaccharide; SST, somatostatin; PBS, phosphate-buffered saline.

Recent studies have suggested that activated microglia are able to produce O_2_^−^ and O_2_^−^ -derived oxidants and they are thought to mediate the loss of nigral DA neurons *in vitro* and *in vivo* (15,16). Hence, the present study investigated whether SST was able to enhance DA neuronal survival by inhibiting LPS-induced production of ROS. Accumulation of ethidium, the fluorescent product of oxidized hydroethidine, was signifi-cantly increased at 48 h in the LPS-treated SN compared with that in the PBS-injected controls, and the LPS-induced oxidant production was dramatically decreased by SST (Fig. 3). These results showed that SST inhibited LPS-induced ROS production.

### SST decreases LPS-induced production of TNF-α, IL-1β and PGE2

**Figure 4:**
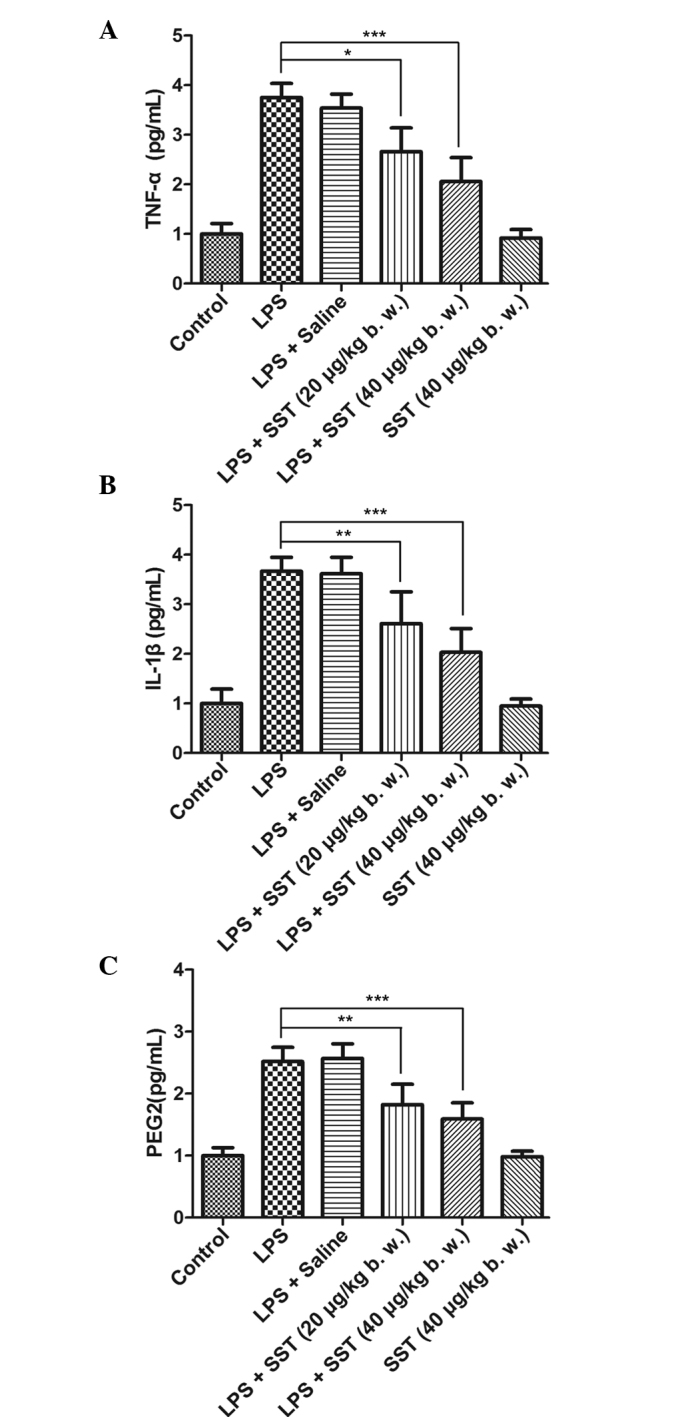
SST reduces the expression of LPS-induced pro-inflammatory mediators. At 24 h after LPS injection, the amounts of (A) TNF-α, (B) IL-1β and (C) PGE2 in the SN were measured using ELISA. Values are presented as the mean ± standard error of the mean of eight animals per group; ^*^P<0.05, **P<0.01 and ^***^P<0.001 compared with the phosphate-buffered saline-injected SN. LPS, lipopolysaccharide; SST, somatostatin; SN, substantia nigra; TNF, tumor necrosis factor; IL, interleukin; PGE, prostaglandin E2.

Several studies have demonstrated that the production of TNF-α, IL-1β and PGE2 are upregulated in LPS-injected SN (14,17). Neuroinflammation is thought to mediate DA neuronal death in the SN (18). Therefore, the present study examined whether SST was able to decrease DA neuronal death by regulating LPS-induced production of TNF-α, IL-1β and PGE2 in the SN. When 20 *μ*g/kg SST was administered prior to LPS injection, the amount of TNF-α, IL-1β and PGE2 was significantly reduced by 24.9% (Fig. 4A), 27.9% (Fig. 4B) and 29.2% (Fig. 4C), respectively. The production of TNF-α, IL-1β and PGE2 was decidedly decreased by 41.8, 43.9 and 41.6%, respectively, when 40 *μ*g/kg SST was administered prior to LPS treatment, while SST had no effect on the production of TNF-α, IL-1β and PGE2 when it was used alone. This confirmed that SST was able to decrease LPS-induced production of TNF-α, IL-1β and PGE2.

### SST decreases LPS-induced overexpression of Cox-2, iNOS and NF-κB p-p65

**Figure 5:**
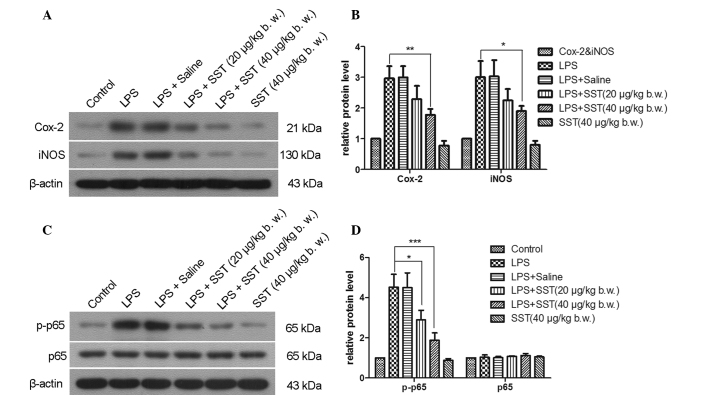
Effect of SST on LPS-induced expression of Cox-2, iNOS and the activation of NF-κB p65. l. (A and C) representative western blot gels showing Cox-2, iNOS, NF-κB p65 and NF-κB p-p65 levels. Cox-2, iNOS and NF-κB p-p65 are represented by the bands at 21, 130 and 65 kDa, respectively. β-actin was used as an internal control. (B and D) Quantification of A and C by gray value analysis. Values are presented as the mean ± standard error of the mean of eight animals per group. ^*^P<0.05, ^**^P<0.01, ^***^P<0.001 compared with phosphate-buffered saline-injected SN. LPS, lipopolysaccharide; SST, somatostatin; SN, substantia nigra; COX, cyclooxygenase; iNOS, inducible nitric oxide synthase; NF-κB, nuclear factor kappa B.

Upregulation of Cox-2 and iNOS have been implicated in DA neuronal cell death of patients with degenerative brain diseases (19,20). Inhibition of the activation of NF-κB p65 was able to prevent DA neuronal cell loss in a 1-methyl-4-phenyl-1,2,3,6-tetrahyropyridine-induced mouse model of PD (21). Accordingly, the present study examined the effect of SST on DA neuronal survival through affecting LPS-induced expression of Cox-2, iNOS and the activation of NF-κB in the SN. 24 hours following injection, LPS enhanced the expression of Cox-2, iNOS and activated NF-κB p65 via phosphorylation (Fig. 5). Administration of 20 *μ*g/kg SST prior to LPS injection reduced the expression of Cox-2, iNOS and NF-κB p-p65 by 23.4, 26.1 and 35.9%, respectively. When 40 *μ*g/kg SST was given prior to LPS injection, the expression of Cox-2, iNOS and NF-κB p-p65 was decreased by 40.5, 37.3 and 58.4%, respectively. SST alone had no effects on Cox-2, iNOS and NF-κB p-p65 expression. It was therefore demonstrated that SST was able to inhibit the expression of Cox-2, iNOS and NF-κB p-p65.

## Discussion

PD is a complex neurodegenerative disorder owing to an aggravating process of neuronal loss within the SN (22). Microglial activation has been reported to induce the death of DA neurons (23). This is due to activated microglia being able to actively produce and secrete unfavorable toxic substances, including pro-inflammatory cytokines and ROS (24). The present study demonstrated that the neuroprotective effects of SST in the LPS-treated SN are based on the ability of SST to suppress microglial activity and thereby decrease the production of pro-inflammatory cytokines and ROS generation. Immunohistochemical staining of OX-42 suggested that SST inhibited microglial activation. Loss of nigral DA cells was decreased by administration of SST prior to LPS injection compared with LPS-treatment only, which was confirmed by immunohistochemical staining for TH and Nissl staining of the SN. Together with the results of a previous study (25), the present study proved that downregulation of microglial activity is able to improve survival of nigral DA neuronal cells. SST is therefore suggested as a potential drug for PD treatment.

Increasing evidence suggested that activated microglia are able to generate ROS, which results in oxidative stress to DA neurons in the SN of PD patients (26). O_2_^−^ and O_2_^−^ -derived oxidant molecules may exacerbate neurotoxicity (27). The accumulation of fluorescent oxidized hydroethidine showed that LPS-induced production of ROS may be mitigated through SST treatment prior to administration of LPS in the SN. Moreover, NO generated by iNOS also contributes to the oxidative stress associated with the neurotoxicity observed in PD (28). Importantly, neurodegeneration has been reported to be associated with up-regulation of iNOS expression in the SN (29). The present study showed that LPS-induced overexpression of iNOS was inhibited by treatment with SST. All these results suggested that LPS-induced activation of microglia and oxidative stress were prevented by SST, resulting in neuroprotection.

Neuroinflammation is an important feature in the progression of neurodegenerative disease (30). The activated microglia expresses pro-inflammatory cytokines including TNF-α, IL-1β and PGE2, which lead to neuronal degeneration in the SN of PD patients (31). The results showed that LPS up regulated production of TNF-α, IL-1β and PGE2 was inhibited by SST application before LPS treatment in the SN. These findings are similar to previous reports that fucoidan significantly inhibits the release of TNF-α and prevents neurotoxicity in LPS-induced rat model of PD (32). It suggests that SST has the ability to inhibit the expression of pro-inflammatory cytokines, and lead to neuroprotection as an inhibitor of neuroinflammation.

In addition, a previous study showed that Cox-2 was rapidly upregulated when inflammation occurred, which appeared to be responsible for the formation of PGE2 and may have contributed to the neurodegenerative process in PD (33). Meanwhile, NF-κB is thought to be a transcriptional controller, which participates in the release of Cox-2, following increased degeneration of DA neurons in an animal model of PD (34). The results of the present study showed that the expression of activated NF-κB p65, Cox-2 and PGE2 in the SN was downregulated when SST was administered prior to LPS treatment compared with that in the group treated with LPS only. The NF-κB/Cox-2/PGE2 signaling pathway was confirmed to participate in the neurodegeneration of the LPS-induced PD model and the inflammatory responses of LPS-treated P12 cells proceed via the same mechanism (17). As suppression of NF-κB is able to attenuate the production of LPS-induced pro-inflammatory mediators (35), the inhibition of NF-κB may be a mechanism underlying the neuroprotective effect of SST. NF-κB, Cox-2 and PGE2 may therefore be considered as targets for PD treatment.

In conclusion, the findings of the present study demonstrated that SST, particularly at high concentrations of SST used in the SN, may inhibit ROS production, expression of pro-inflammatory cytokines and the NF-κB pathway as well as the activation of microglia, which may lead to increased neuronal survival. These results suggest that SST offers great therapeutic potential for treating neurodegenerative diseases, such as PD, through inhibiting microglial activation.
